# Identification and Expression Analysis of the Soybean Serine Acetyltransferase (*SAT*) Gene Family Under Salt Stress

**DOI:** 10.3390/ijms26051882

**Published:** 2025-02-22

**Authors:** Caiyun Fan, Hui Zou, Miao Zhang, Yu Jiang, Baohui Liu, Zhihui Sun, Bohong Su

**Affiliations:** 1 Guangzhou Key Laboratory of Crop Gene Editing, Guangdong Key Laboratory of Plant Adaptation and Molecular Design, Innovative Center of Molecular Genetics and Evolution, School of Life Sciences, Guangzhou University, Guangzhou 510006, China; 2 College of Agriculture, China Agricultural University, Beijing 100080, China; b20243010023@cau.edu.cn

**Keywords:** soybean, *SAT* gene family, cis-regulatory elements, salt stress, expression analysis

## Abstract

Serine acetyltransferase (SAT) is a critical enzyme in the sulfur-assimilation pathway of cysteine, playing an essential role in numerous physiological functions in plants, particularly in their response to environmental stresses. However, the structural characteristics of the soybean *SAT* gene family remain poorly understood. Members of the soybean *SAT* gene family were identified using the Hidden Markov Model approach. Bioinformatics tools, such as ExPASy, PlantCARE, MEME, and TBtools-II, were employed to examine the physicochemical properties, cis-regulatory elements, conserved motifs, gene structures, and chromosomal positions of the *GmSAT* genes. RT-qPCR was conducted to evaluate the expression profiles of *GmSAT* genes under NaCl-induced stress, identifying genes likely involved in the salt-stress response. A total of ten *GmSAT* genes were identified in the soybean genome and grouped into three subfamilies. Genes within each subfamily shared notable structural similarities and conserved motifs. Analysis of cis-regulatory elements revealed that the promoters of these genes contain several elements linked to plant growth and stress-related responses. Expression patterns of *GmSAT* genes varied across different soybean tissues, with *GmSAT10* showing higher expression in roots, while *GmSAT1* and *GmSAT2* had lower expression in the same tissue. Following NaCl treatment, expression levels of seven *GmSAT* genes were significantly increased in the roots, indicating their potential involvement in the plant’s adaptation to salt stress. *GmSAT* genes appear to play crucial roles in soybean’s response to salt stress, offering insights that could aid in the development of salt-tolerant soybean varieties.

## 1. Introduction

Sulfur is an essential macronutrient required for plant growth and development, constituting approximately 0.3–0.5% of the total dry weight in plants, making it the fourth most abundant element after nitrogen, phosphorus, and potassium [1]. Plants, microorganisms, and fungi acquire sulfur primarily from inorganic sulfates in the soil, as well as from atmospheric derivatives of sulfites or sulfides [2]. In most organisms, serine acetyltransferase (SAT) is the key initiating enzyme in the cysteine (Cys) biosynthesis pathway. Cysteine is a multifunctional sulfur-containing non-essential amino acid involved in the synthesis of proteins, glutathione (GSH), vitamins, thioesters, coenzyme A, and taurine [3]. The biosynthesis of cysteine begins with the acetylation of serine by SAT, which catalyzes the transfer of an acetyl group from acetyl-CoA to serine, forming O-acetylserine (OAS). OAS serves as the key precursor for cysteine synthesis. In the next step, O-acetylserine (thiol) lyase (OASTL) catalyzes the displacement of the acetyl group by hydrogen sulfide (H_2_S), leading to the production of cysteine [4,5,6]. Although SAT and OASTL catalyze two distinct enzymatic steps in the cysteine biosynthetic pathway, they are often assembled into a functional cysteine synthase complex (CSC). This complex is composed of two SAT homotrimers and two OASTL homodimers, which cooperate in a highly coordinated manner to facilitate the efficient regulation of cysteine synthesis. Within the CSC, SAT retains its catalytic activity, whereas it is enzymatically inactive in its unbound form. In contrast, OASTL displays enzymatic activity exclusively in its free state [3].

Serine acetyltransferase contains two key functional domains: the SATase_N domain and the Hexapep_C domain, both of which exhibit a high degree of conservation [7,8]. The SAT protein sequence contains two distinct regions involved in protein–protein interactions: a central homodimerization domain that mediates SAT-SAT interactions and a C-terminal heterodimerization domain responsible for SAT-OASTL interactions [8]. The Arabidopsis *SAT* genes family includes five members: *AtSERAT1; 1* (*AtSAT5*), *AtSERAT2; 1* (*AtSAT1*), *AtSERAT2; 2* (*AtSAT3*), *AtSERAT3; 1* (*AtSAT2*), and *AtSERAT3; 2* (*AtSAT4*) [9]. AtSAT2, AtSAT4, and AtSAT5 are localized in the cytosol, AtSAT1 is localized in plastids, and AtSAT3 is localized in the mitochondria. Among these isoforms, *AtSAT1*, *AtSAT3*, and *AtSAT5* are considered to play pivotal roles in the biosynthesis of OAS [10].

As the rate-limiting enzyme in the biosynthesis of Cys, SAT is of vital importance in multiple aspects of plants. It significantly influences plant growth and development processes, and it also determines how plants respond to abiotic stress. In *Arabidopsis thaliana*, single *SAT* gene knockout mutants do not exhibit significant phenotypic alterations. However, mutants with a knockout of four *SAT* genes are viable but display slowed growth, while the complete knockout of all five *SAT* genes results in embryo lethality. These findings suggest functional redundancy among *SAT* gene family members, while also underscoring the essential role of the *SAT* gene family in plant viability [11]. Overexpression of Arabidopsis *SAT* gene in tobacco leaves increases cysteine content, whereas overexpression of maize *SAT* gene in endosperm tissue leads to zein accumulation without impairing normal plant growth [12,13].

Soil salinization is a global agricultural issue that severely threatens crop yield and quality [14,15]. Soybean (*Glycine max* (Linn.) Merr.), a crucial food and oilseed crop, accounts for over 50% of global soybean oil production and provides more than 25% of plant-based protein, holding significant importance in the global agricultural economy [16,17]. Soybean is considered a moderately salt-tolerant crop with a relatively short growth cycle [18]. However, cultivating soybeans in saline-alkaline soils inhibits their growth and development, resulting in reduced yield [19]. Under salt stress, soybean seed germination is suppressed, and the accumulation of key nutrients, such as proteins, lipids, and fatty acids, is adversely impacted, which lowers both the nutritional value and processing quality of the crop [20]. Therefore, enhancing soybean salt tolerance can improve its adaptability to environmental stresses, enabling cultivation in low-salt or alkaline soils, thus expanding the soybean cultivation area and boosting yield.

Studies have shown that certain *SAT* genes in plants, such as *Arabidopsis thaliana* [8], *Solanum lycopersicum* [21], and *Camellia sinensis* [22], are involved in the response to salt stress. A notable feature of plant responses to abiotic stress is the production of reactive oxygen species (ROS), and excessive accumulation of ROS can lead to oxidative damage, which, in severe cases, results in programmed cell death [23]. When plants are exposed to stresses such as heavy metals, drought, or salinity, the sulfur metabolism pathway is activated, leading to changes in the expression and activity of serine acetyltransferase. This regulation controls the synthesis of antioxidant compounds, such as cysteine and glutathione. This process helps reduce ROS accumulation, enhances salt tolerance, and improves the plant’s overall stress resistance [24,25,26]. However, up to now, no studies have been published regarding the response of the soybean *SAT* gene family to salt stress.

The *SAT* gene family has been identified in multiple plant species, with Arabidopsis harboring five *SAT* genes [8], rice possessing six *SAT* genes [27], tomato containing four *SAT* genes [21], and tea plants exhibiting seven *SAT* genes [22]. In this study, bioinformatics approaches were employed to identify ten *SAT* gene family members within the soybean genome. Comprehensive analysis was performed to assess their physicochemical properties, chromosomal localization, phylogenetic relationships, gene structures, conserved motifs, intraspecific synteny, cis-regulatory elements, and gene expression patterns across various tissues. Additionally, RT-qPCR was utilized to investigate the expression of *GmSAT* genes in soybean leaves and roots under salt stress, thereby elucidating the role of the soybean *SAT* gene family in the salt-stress response. The findings of this study offer a theoretical foundation for further investigation into the soybean *SAT* gene family, the identification of salt-tolerance-related genes, and the development of molecular breeding strategies for improving salt tolerance in soybean.

## 2. Results

### 2.1. Characterization and Physicochemical Analysis of Soybean SAT Gene Family Members

To identify *SAT* gene family members in soybean, a BLAST search was conducted using the SAT protein sequences from *Arabidopsis thaliana* as reference sequences, leading to the identification of 10 members in *Glycine max.* (Table S1). These genes were subsequently mapped to soybean chromosomes and sequentially named based on their chromosomal positions, designated as *GmSAT1* through to *GmSAT10*. To further explore the physicochemical properties of these genes, the amino acid sequences of all GmSAT proteins were assessed through the ExPASy database. The results revealed considerable variability in the physicochemical characteristics of the soybean *SAT* gene family. The encoded proteins ranged in size from 286 to 404 amino acids, with molecular weights between 30.36 kDa and 44.29 kDa. The isoelectric points (pI) of the proteins varied from 5.71 to 8.52, and their instability indices ranged from 30.52 to 44.98. Subcellular localization predictions indicated that one SAT protein is localized to the nucleus, six to the chloroplast, and three to the cytoplasm. These findings suggest that the soybean *SAT* genes may play diverse roles in various cellular compartments (Table S1).

### 2.2. Phylogenetic Tree Construction of the Soybean SAT Gene Family

**Figure 1:**
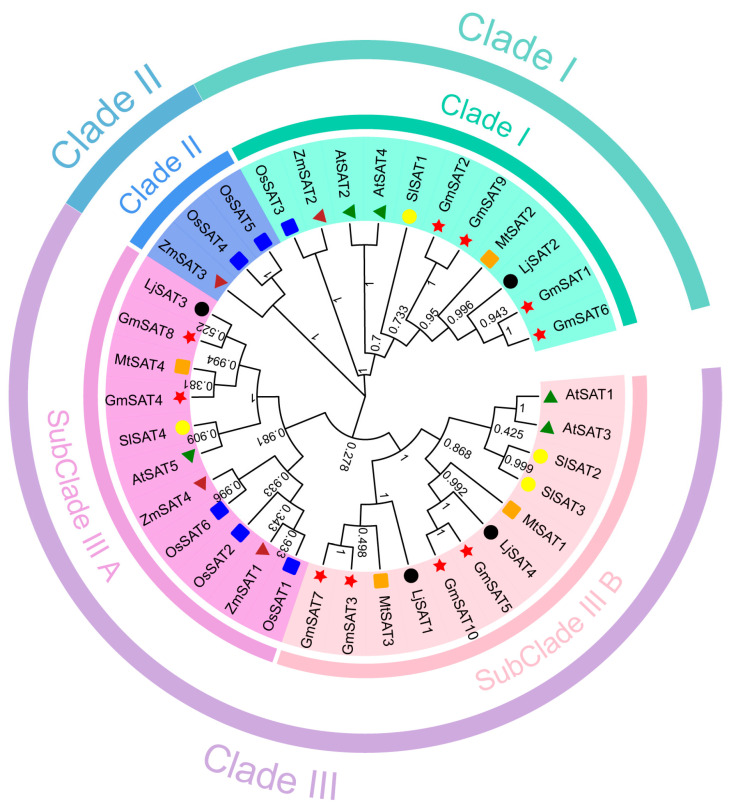
Phylogenetic tree depicting the evolutionary relationships of *SAT* genes across dicot and monocot species. The phylogenetic tree was constructed by aligning SAT protein sequences from a diverse range of plant species, and the tree topology was inferred using the Neighbor-Joining (NJ) method for clustering. SAT family proteins from different species are represented by distinct symbols: red pentagrams for *Glycine max* (*Gm*), green triangles for *Arabidopsis thaliana* (*At*), black circles for *Lotus japonicus* (*Lj*), orange squares for *Medicago truncatula* (*Mt*), yellow circles for *Solanum lycopersicum* (*Sl*), blue squares for *Oryza sativa* (*Os*), and brown triangles for *Zea mays* (*Zm*). Clades I–III are color-coded to indicate the evolutionary groupings of the SAT proteins.

To explore the evolutionary relationships among SAT proteins in soybean and other species, protein sequences from *Glycine max*, *Oryza sativa*, *Lotus japonicus*, *Medicago truncatula*, *Solanum lycopersicum*, *Zea mays*, and *Arabidopsis thaliana* were used to construct a phylogenetic tree. Evolutionary analysis of the soybean *SAT* gene family identified three primary clades: Clade I, Clade II, and Clade III, with the latter further divided into SubClade III A and SubClade III B. Clade I includes four soybean *SAT* genes, SubClade III A includes two soybean *SAT* genes, and SubClade III B contains four soybean *SAT* genes. Phylogenetic branching clearly separated *SAT* genes from monocotyledons and dicotyledons into distinct groups. For example, within Clade I, *SAT* genes from dicotyledons (*GmSAT1*, *GmSAT2*, *GmSAT6*, *GmSAT9*, *AtSAT2*, *AtSAT4*, *LjSAT2*, *MtSAT2*, *SlSAT1*) formed one subgroup, while *SAT* genes from monocotyledons (*OsSAT3*, *ZmSAT2*) formed a separate subgroup (Figure 1). Phylogenetic analysis indicates that the soybean *SAT* genes share close evolutionary relationships with *SAT* genes from *AtSATs*, *LjSATs*, *MtSATs*, *SlSATs*, *OsSATs*, and *ZmSATs*, suggesting a conserved evolutionary process of the *SAT* gene family across species. Taxonomically, *SAT* gene family members in the Fabaceae, Poaceae, Brassicaceae, and Solanaceae families were clearly distinguished, indicating potential diversity and functional divergence in their evolutionary trajectories.

### 2.3. Chromosomal Localization of the Soybean SAT Gene Family

**Figure 2:**
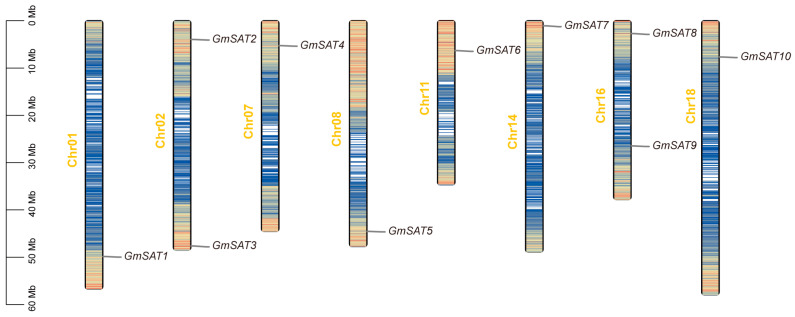
Chromosomal localization of the soybean *SAT* gene family. The scale on the left represents the length of soybean chromosomes in megabases (Mb). Gene density is depicted in a color gradient, with red indicating regions of higher gene density and blue indicating regions of lower gene density.

The ten members of the *GmSAT* gene family are unevenly distributed across eight distinct chromosomes: Chr01, Chr02, Chr07, Chr08, Chr11, Chr14, Chr16, and Chr18 (Figure 2). Notably, multiple copies of the *SAT* gene were observed on several chromosomes, with Chr02 and Chr16 each containing two *GmSAT* genes, while the remaining chromosomes harbor a single *GmSAT* gene. Specifically, *GmSAT1*, *GmSAT3*, *GmSAT5*, and *GmSAT9* are located on the lower ends of their respective chromosomes, whereas *GmSAT2*, *GmSAT4*, *GmSAT6*, *GmSAT7*, *GmSAT8*, and *GmSAT10* are positioned on the upper ends. These findings suggest a dispersed distribution pattern of the *GmSAT* genes across the soybean genome.

### 2.4. Intraspecies Collinearity Analysis of GmSAT Genes

**Figure 3:**
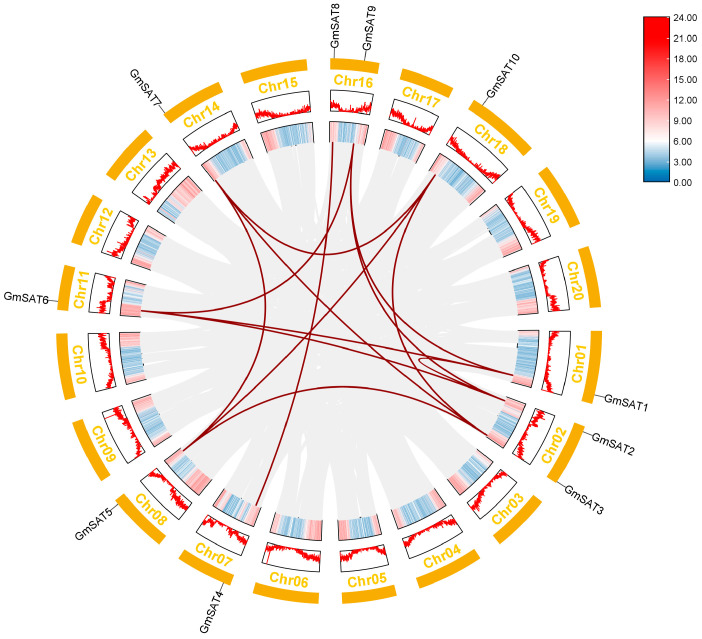
Intraspecies collinearity analysis of the *GmSAT* gene family. The 20 soybean chromosomes are depicted as orange rectangles, with the corresponding chromosome numbers indicated at the top of each rectangle. The GC content and gene density across the chromosomes are visualized using different colored lines and heatmaps, respectively. Gray lines represent the connections between all duplicated sequences within the soybean genome, while red lines specifically highlight the segmental duplication pairs of *GmSAT* genes.

Intraspecies collinearity analysis of the *GmSAT* gene family was performed using TBtools-II, employing the MCScanX algorithm. This method is designed to identify and visualize collinear regions within the genome, thus revealing conserved genomic segments within a species. Gene duplication events, which are central to genome evolution, play a crucial role in the diversification of gene functions and the amplification of gene families [28]. These duplications can occur through segmental or tandem duplications [29]. To gain deeper insights into the evolutionary dynamics of the soybean *SAT* gene family, intraspecies synteny analysis was performed. The results confirmed the presence of segmental duplications, but no tandem duplications, among the soybean *SAT* genes. A total of 13 segmental duplication events involving *GmSAT* genes were detected across the soybean genome sequence (Figure 3). These results indicate that the expansion of the *SAT* gene family in soybean can largely be attributed to segmental duplications.

### 2.5. Structure, Conserved Motifs, and Conserved Protein Domains of GmSAT Genes

**Figure 4:**
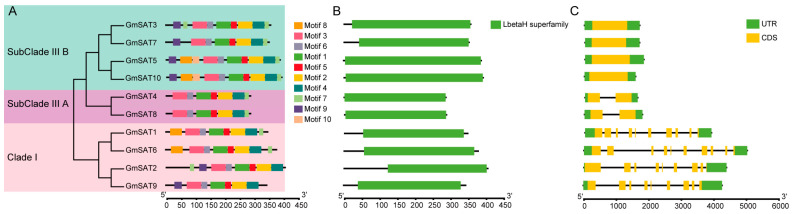
Structural features of *SAT* gene family members. (**A**) Phylogenetic relationships and distribution of conserved motifs within the *GmSAT* gene family. The conserved motifs in the 10 GmSAT proteins were determined using the MEME tool. Non-conserved regions are represented by black lines, while each conserved motif is indicated by a colored box as shown on the right. The scale at the bottom illustrates the relative lengths of each motif within the protein sequences. (**B**) Conserved protein domains within the SAT proteins. The full-length protein sequences are represented, with conserved regions highlighted by green boxes. (**C**) Exon–intron organization of the *SAT* genes. Untranslated regions (UTRs) are marked with green boxes, coding sequences (CDS) are indicated by yellow boxes, and introns are represented by black lines. The scale at the bottom shows the proportional lengths of exons and introns.

To further investigate the conserved motif composition of GmSAT proteins, motif prediction was conducted using MEME online suite. Ten conserved motifs (Motifs 1–10) were detected across the 10 GmSAT proteins (Figure 4A), with detailed sequence information for each motif presented in Figure S1. Motifs 1–6 were conserved in all 10 GmSAT proteins, while Motif 10 was found only in two members. Most conserved motifs were localized in the C-terminal regions of the proteins, and paralogous members within the same clade exhibited similar types and arrangements of motifs. For example, GmSAT5 and GmSAT10 contained the highest number of motifs, with all 10 motifs present in both proteins. Each GmSAT protein contained between 7 and 10 motifs. Moreover, all proteins encoded by the *GmSAT* genes were found to possess a conserved left-handed parallel β-helix (LβH) domain, a structural motif typically associated with acyltransferase activity (Figure 4B). To assess both the structural conservation and diversity within the *GmSAT* gene family, structural models for all 10 GmSAT proteins were generated (Figure 4C). Gene structure analysis revealed significant variability in the intron count, ranging from 0 to 9. Specifically, six *GmSAT* genes contained only 1–2 exons, while the four members of Clade I included 8–9 exons. This variation indicates structural diversity within the gene family. Within the same clade, members exhibited similar exon–intron structural patterns, particularly in terms of exon number and length, suggesting a high degree of conservation. However, significant differences in exon number and length were observed between clades, likely resulting from genomic polymorphisms or mutations. These differences may reflect functional diversification during evolution. Analysis of conserved motif distribution, gene structure, and conserved protein domains revealed that members within the same evolutionary clade were highly similar, whereas those in different clades displayed substantial differences. These findings suggest functional divergence among *GmSAT* genes in distinct evolutionary branches, potentially reflecting differences in their biological roles or regulatory mechanisms. These findings deepen our understanding of the structural variability and functional heterogeneity present within the *GmSAT* gene family.

### 2.6. Cis-Acting Element Analysis of Promoters

**Figure 5:**
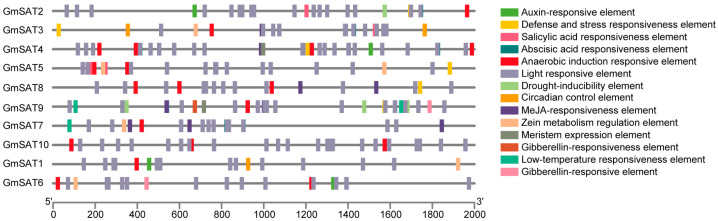
Analysis of Cis-acting Elements in the Promoters of the Soybean *SAT* Gene Family. Different categories of cis-acting elements are depicted using color-coded boxes. The promoter sequence lengths are represented by the scale at the bottom, providing a reference for the relative sizes.

Cis-regulatory elements located within promoter regions play a pivotal role in the activation and modulation of gene transcription. To explore the diversity and spatial arrangement of cis-regulatory elements in the promoter regions of *GmSAT* genes, the 2000 bp upstream sequences of each member of the soybean *SAT* gene family were extracted using TBtools-II v2.111 for cis-regulatory element prediction. Following the prediction, 14 distinct cis-regulatory elements were identified for subsequent functional characterization (Figure 5, Table S2). This analysis demonstrates that all *GmSAT* gene family members harbor a broad range of cis-regulatory elements within their promoter regions. Among these, light-responsive elements were particularly abundant across all family members, suggesting their potential role in the regulation of gene expression in response to light (Figure S2). Furthermore, the promoter regions were found to contain cis-acting elements responsive to plant hormones, including salicylic acid, abscisic acid, auxin, and gibberellins. Additionally, elements associated with responses to environmental stressors, such as anaerobic conditions, drought, low temperature, and circadian rhythms, were also present. These findings indicate that *GmSAT* genes may play a significant role in regulating stress responses and hormone signaling in soybean, highlighting their potential involvement in diverse physiological processes.

### 2.7. Expression Patterns of GmSAT Genes in Different Tissues

**Figure 6:**
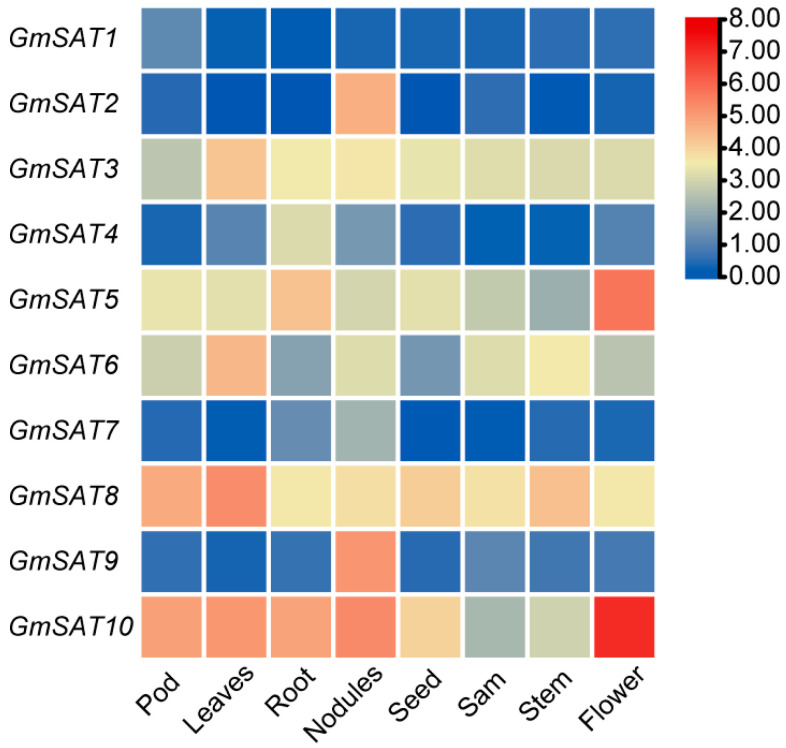
Expression analysis of *SAT* genes in different tissues. The FPKM values of *GmSAT* genes were transformed using a log2 scale. The expression levels across different tissues are represented by a color gradient, with low expression depicted in blue and high expression in red, as indicated by the bar chart on the right.

To explore the expression profiles of the *SAT* gene family in various soybean tissues and organs, transcriptomic data for the *GmSAT* genes in eight distinct soybean tissues were retrieved from the Phytozome 13 database (Figure 6, Table S3). The analysis uncovered tissue-specific expression patterns of *GmSAT* genes. Specifically, *GmSAT8* and *GmSAT10* were expressed across all tissues, exhibiting notably elevated expression levels, while *GmSAT1*, *GmSAT2*, and *GmSAT7* showed the lowest expression levels. Remarkably, *GmSAT10* displayed the highest expression in flowers and the lowest in shoot apical meristems. Conversely, *GmSAT2* and *GmSAT9* were most abundantly expressed in nodules, with reduced expression observed in flowers. These observations imply that individual *GmSAT* genes may play tissue-specific roles, emphasizing the functional diversity of the *SAT* gene family in different soybean tissues.

### 2.8. Expression of SAT Genes Under Salt Stress

**Figure 7:**
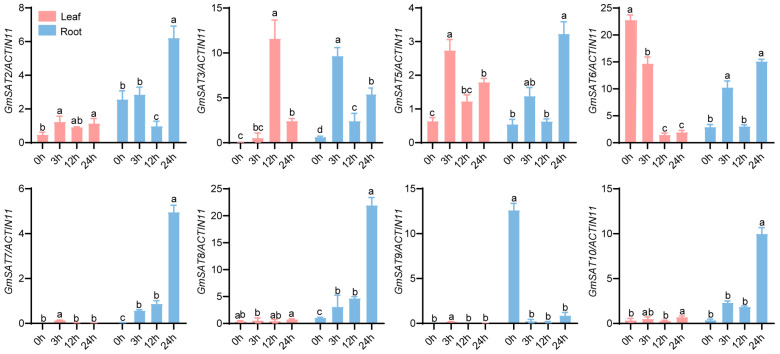
RT-qPCR analysis of the expression of eight *GmSAT* genes under salt stress. The baseline measurement at 0 h was designated as the control, with *Gmactin11* serving as the internal reference gene for normalization. Error bars represent the standard deviation calculated from three independent biological replicates. Statistical significance was determined through one-way analysis of variance (ANOVA), followed by Duncan’s multiple range test (*p* < 0.05, n = 3). Different letters denote statistically significant differences between groups. The pink color in the figure represents soybean leaves, while blue indicates soybean roots.

To investigate the expression patterns of *GmSAT* genes in response to salt stress, soybean leaves and roots were collected at different time points (0 h, 3 h, 12 h, and 24 h) following salt treatment (200 mM NaCl), and the expression levels of eight *GmSAT* genes were analyzed using RT-qPCR (Figure 7). Compared to other *GmSAT* genes, the expression of *GmSAT7*, *GmSAT8*, *GmSAT9*, and *GmSAT10* in leaves was consistently the lowest across all salt treatments. In roots, *GmSAT7*, *GmSAT8*, and *GmSAT10* exhibited similar expression patterns in response to salt stress, with expression levels increasing at 3 h, 12 h, and 24 h of NaCl treatment, reaching their highest levels at 24 h. These results suggest that these genes may play a role in the later stages of the plant’s adaptation to salt stress. In contrast, *GmSAT9* showed a significant decrease in expression in roots under salt stress, while *GmSAT6* expression in leaves significantly decreased, potentially indicating a negative regulatory mechanism in response to salt stress. *GmSAT2*, *GmSAT3*, *GmSAT5*, and *GmSAT6* exhibited similar expression patterns in roots, with expression levels increasing at 3 h, decreasing significantly at 12 h, and then increasing again at 24 h. Both *GmSAT2* and *GmSAT5* exhibited comparable expression profiles in both the aerial and subterranean tissues, indicating that these genes could potentially perform similar functions in mediating salt tolerance across both above-ground and below-ground plant structures.

## 3. Discussion

*SAT* genes are critical components of the sulfur-assimilation pathway leading to cysteine biosynthesis and are widely distributed across various organisms, including bacteria, fungi, and plants [30]. In this study, we identified 10 *GmSAT* genes through a comprehensive analysis of the soybean genome, which is notably higher than the number of *SAT* gene family members found in other species, such as Arabidopsis (5), rice (6), and tomato (4) [8,21,27]. This disparity may be attributed to differences in genome size among species, suggesting that soybean has undergone a more complex evolutionary history within the *GmSAT* gene family. In addition to possessing a larger number of *GmSAT* genes, soybean exhibits a widespread distribution of these genes across multiple chromosomes. This distribution pattern may contribute to the enhancement of genetic diversity and stability. The primary mode of *GmSAT* gene duplication in soybean is segmental, with 13 segmental duplication events identified, underscoring the critical role of segmental duplication in the expansion of the *GmSAT* gene family. The expansion of these gene family members could result in functional redundancy or specificity among different members, thereby facilitating the fine-tuned regulation of various biological processes.

The characteristics of SAT enzymes are defined by the presence of two highly conserved structural domains: SATase_N (PF06426) and Hexapep_C (PF00132), both of which exhibit significant conservation across different plant species [31]. The SATase_N domain plays a crucial role in catalyzing the reaction between serine and acetyl-CoA to produce O-acetylserine (OAS), while the Hexapep_C domain is essential for the formation of the cysteine synthase complex. In this study, we found that the soybean *SAT* gene family contains both of these conserved domains. Additionally, the C-terminal region of SAT proteins exhibits substantial conservation, and mutations at key amino acid residues in this region result in a significant reduction in enzymatic activity [32,33]. These findings reinforce the functional importance of these conserved domains in the enzymatic activity and structural integrity of SAT proteins.

From the perspective of gene structure, members of the soybean *GmSAT* gene family exhibit considerable variation. Specifically, *GmSAT1* and *GmSAT6* contain 9 introns; *GmSAT2* and *GmSAT9* have 8 introns; *GmSAT4* and *GmSAT8* possess 1 intron; and *GmSAT3*, *GmSAT5*, *GmSAT7*, and *GmSAT10* are intronless. This structural diversity is consistent with the findings of previous research in rice, where the gene structures of the *OsSAT* gene family members also exhibit considerable variation. Notably, except for *OsSAT3*, which contains 10 exons, the other *OsSAT* genes have only 1 exon [27]. In our study, four *GmSAT* genes were found to consist of a single exon without any introns. Previous studies suggest that genes with few or no introns tend to exhibit rapid expression in response to biotic and abiotic stresses [34,35]. This characteristic may contribute to the swift transcriptional response of *GmSAT* genes under stress conditions. Furthermore, the conserved motif numbering and the distribution of these motifs across the soybean *SAT* genes indicate that the SAT domain is highly conserved in soybean, reinforcing the functional importance of this conserved region in the *GmSAT* gene family.

This investigation characterized various cis-regulatory elements within the promoter regions of the soybean *SAT* gene cluster, including those responsive to light, plant hormones, and stress signals. The findings suggest that *GmSAT* genes may be modulated by light stimuli and hormonal cues, or they may participate in the signaling networks associated with these factors. The tea plant *SAT* gene family, similar to the distribution pattern of promoter cis-acting elements observed in soybean, contains various promoter cis-acting elements, including those associated with jasmonate and low-temperature response. These elements play a crucial role in regulating a range of physiological processes in the tea tree, such as stress response and hormone signaling [22]. Additionally, the identification of stress-responsive motifs suggests that the *GmSAT* genes may play a pivotal role in regulating processes essential for the growth, maturation, and stress resilience of soybean. However, to comprehensively understand the precise mechanisms of action, further experimental investigations are necessary to delineate the specific functions of the *GmSAT* gene family in response to both biotic and abiotic stressors, as well as to advance our knowledge of their contributions to stress adaptation and resistance pathways.

*SAT* genes play a crucial role in plant growth and development. In Arabidopsis, knockout mutants of a single *SAT* gene do not exhibit significant phenotypic changes, but the *atsat-5m* mutant leads to plant death, highlighting the essential role of SAT in plant growth and development, while also suggesting functional redundancy among *SAT* genes [11]. Through database analysis, we observed that *GmSAT* gene members exhibit tissue- and organ-specific expression patterns. Among these, *GmSAT10* exhibits the highest overall relative expression, indicating its potential involvement across multiple tissues and organs. Notably, *GmSAT5* and *GmSAT10* demonstrate markedly elevated expression in floral tissues relative to other organs, whereas *GmSAT2* is predominantly expressed within root nodules. These expression patterns imply that *GmSAT* genes may play distinct roles in flower development, biological nitrogen fixation, and other physiological processes in soybean. The observed differences in the expression patterns of various *SAT* gene family members across different soybean tissues suggest functional differentiation, providing a solid foundation for further investigation into the roles of *GmSAT* genes in soybean growth and development.

Studies have shown that *OsSAT1; 1*, *OsSAT1; 2*, and *OsSAT1; 3* are upregulated at different stages of salt stress [27]. Similarly, under 24 h of salt stress, chloroplast SAT activity in tomato was significantly increased, accompanied by a notable upregulation of the transcription level of *SlSAT2; 2* [21]. In this study, RT-qPCR analysis revealed differential expression patterns of *GmSAT* gene family members under salt-stress conditions. Notably, the expression levels of *GmSAT7*, *GmSAT8*, and *GmSAT10* were significantly upregulated in soybean roots under prolonged salt stress, indicating that these genes could be implicated in the molecular mechanisms underlying the plant’s response to saline conditions. These results offer valuable insights into the possible involvement of *GmSAT* genes in stress resilience, underscoring the necessity for further investigations to clarify their precise roles in soybean adaptation to salinity stress.

## 4. Materials and Methods

### 4.1. Materials

In this study, the widely used soybean cultivar “Williams 82” (*Glycine max* Wm82.a4.v1) was selected as the experimental material as its genome sequence is well-annotated. All seeds were sourced from the germplasm repository of our laboratory, ensuring the reliability and consistency of the seed source and quality [36]. Prior to sowing, soybean seeds with uniform size and full development were selected. The seeds were then disinfected with 75% ethanol for 3 min, followed by five washes with sterile water to remove surface microbes and impurities.

### 4.2. Identification of GmSAT Gene Family and Prediction of Protein Physicochemical Properties

To identify the members of the soybean *SAT* gene family, genome sequences, amino acid sequences, and genome annotation files were downloaded from the Phytozome 13 database (https://phytozome-next.jgi.doe.gov/, accessed on 15 June 2024) [37] and the Ensembl Plants database (http://plants.ensembl.org/, accessed on 15 June 2024) [38]. Two methods were combined to identify the members of the *GmSAT* gene family. First, the query sequence *AtSAT2;1* (*AT1G55920*) was obtained from the published literature [8]. The protein sequences of the *Arabidopsis SAT* family were downloaded from the TAIR database (https://www.arabidopsis.org/, accessed on 15 June 2024) and used for BLAST searches in the Phytozome 13 database (https://phytozome-next.jgi.doe.gov/, accessed on 15 June 2024) and the SoyBase database (https://www.soybase.org/, accessed on 15 June 2024) to identify potential soybean *SAT* genes. Additionally, the Hidden Markov Model (HMM) PF06426, used for identifying *SAT* genes, was downloaded from the Pfam database (http://pfam.xfam.org/, accessed on 15 June 2024) [39], and HMM-based searches were conducted using TBtools-II v2.111 bioinformatics software to identify soybean *SAT* genes [40]. The results from both methods were merged to identify the members of the soybean *SAT* gene family. The conserved domains of these SAT proteins were analyzed using the online CDD program (https://www.ncbi.nlm.nih.gov/Structure/cdd/wrpsb.cgi, accessed on 15 June 2024). A total of ten soybean *SAT* gene family members were identified. The physicochemical properties of the GmSAT proteins, including molecular weight, amino acid count, isoelectric point, and instability index, were analyzed using the ProtParam tool from the ExPASy online database (https://www.expasy.org/, accessed on 20 June 2024) [41]. Subcellular localization predictions for each soybean SAT protein were made using WoLF PSORT (https://www.genscript.com/wolf-psort.html, accessed on 20 June 2024) to provide insights into their potential cellular localization.

### 4.3. Phylogenetic Analysis of the SAT Gene in Soybean

Protein sequences containing conserved SAT domains from *Glycine max*, *Oryza sativa*, *Zea mays*, *Solanum lycopersicum*, *Medicago sativa*, *Lotus japonicus*, and *Arabidopsis thaliana* were retrieved from the Phytozome 13 database (https://phytozome-next.jgi.doe.gov/, accessed on 3 July 2024) and TAIR database (https://www.arabidopsis.org/, accessed on 3 July 2024). Sequence alignment was performed using ClustalW 2.1 [42] implemented in MEGA7.0 software [43]. A phylogenetic tree was constructed using the neighbor-joining (NJ) method with 1000 bootstrap replications, while all other parameters were set to their default values. The resulting tree file (nwk format) was further refined and visualized using the online tool Evolview (https://www.evolgenius.info/, accessed on 5 July 2024) [44].

### 4.4. Chromosomal Localization of Soybean SAT Gene Family Members

To analyze the distribution of these genes, the Gene Density Profile tool in TBtools-II v2.111 was used to visualize the chromosome mapping, utilizing the GFF3 annotation file downloaded from Phytozome 13 and the positional data of the 10 *GmSAT* gene family members.

### 4.5. Intraspecies Collinearity Analysis

The whole-genome sequence and gene annotation files for soybean were obtained from Phytozome. Collinearity analysis was conducted using TBtools-II v2.111 software, and the results were visualized to generate a collinearity map illustrating the relationships among the *GmSAT* genes.

### 4.6. Gene Structure and Conserved Motifs

The gene structures of the *SAT* gene family were visualized using TBtools-II v2.111, based on the GFF3 annotation file. Conserved motif prediction for the soybean *SAT* gene family members was performed using the MEME online suite (https://meme-suite.org/meme/, accessed on 26 July 2024) [45], with the number of motifs set to 10 and all other parameters maintained at their default settings. The results were subsequently visualized and plotted using TBtools-II v2.111 [40].

### 4.7. Promoter Cis-Acting Element Analysis

The upstream 2000 bp nucleotide sequences of the CDS regions for each member of the soybean *SAT* gene family were extracted using TBtools-II v2.111 and designated as promoter sequences. These sequences were then analyzed and predicted for cis-acting elements using the PlantCARE online tool (http://bioinformatics.psb.ugent.be/webtools/plantcare/html/, accessed on 12 August 2024). The results were visualized and refined using TBtools-II v2.111 to generate a map depicting the identified cis-regulatory elements.

### 4.8. Expression Pattern Analysis of GmSAT Gene Family

Transcriptomic data representing the expression levels of the 10 *GmSAT* gene family members in various tissues were retrieved from Phytozome 13. Heatmaps depicting the expression patterns of the *GmSAT* genes were generated using the Heat Map function in TBtools-II v2.111 software.

### 4.9. Expression Analysis of Soybean SAT Gene Family Members Under Salt Stress

Clean soybean seeds were sown in moist vermiculite and grown in a growth chamber under controlled conditions: a constant temperature of 25 °C, relative humidity of 70%, and long-day photoperiod (16 h light/8 h dark). After approximately 5 days, the seedlings were transferred to tanks containing Hoagland nutrient solution and allowed to grow until reaching the V1 developmental stage. The plants were then divided into two groups: the experimental group was treated with nutrient solution containing 200 mM/L NaCl, while the control group was maintained in nutrient solution without NaCl. During cultivation, continuous aeration was provided via an oxygen pump to ensure sufficient oxygen supply in the nutrient solution for optimal root respiration. RNA was extracted from soybean leaves and roots using the FastPure Universal Plant Total RNA Isolation Kit (RC411-01, Vazyme-innovation in enzyme technology, Nanjing, China), and cDNA was synthesized using the HiScript III RT SuperMix for qPCR kit (R323-01, Vazyme). RT-qPCR reactions were performed in a total volume of 10.0 μL, containing 5.0 μL SYBR Green master mix (Q321-02, Vazyme), 3.6 μL RNase-free water, 0.2 μL of each forward and reverse primer, and 1.0 μL of cDNA template. PCR amplification was carried out using a qTOWER^3^G real-time PCR system (Analytik Jena GmbH, Jena, Germany). Three independent biological replicates were conducted for each treatment. *Gmactin11* was used as an internal reference gene [46]. Primer sequences used in this study are listed in Table S4. We utilized the 2^−∆∆Ct^ method to compute the relative expression levels of the genes [47].

## 5. Conclusions

In this study, we identified 10 *GmSAT* genes, which are distributed across eight different chromosomes in the soybean genome. Phylogenetic analysis categorized these genes into three distinct subgroups, each exhibiting highly conserved motifs and similar gene structural characteristics. *SAT* genes within the same evolutionary branch displayed notable similarities, suggesting that they may share functional roles. In addition, the analysis of cis-regulatory elements and gene expression indicates that the soybean *SAT* gene family may be involved in regulating key processes related to plant hormones, light responses, and biotic stresses, highlighting their important role in adapting to environmental changes. Phylogenetic and synteny analyses provided insights into the homologous relationships of soybean *SAT* genes both within the species and across different plant species, enhancing our understanding of their evolutionary mechanisms. Additionally, RT-qPCR results revealed that *GmSAT7*, *GmSAT8*, and *GmSAT10* are significantly upregulated in roots under salt-stress conditions, suggesting their pivotal role in the plant’s response to salt stress. This study offers a comprehensive analysis of the soybean *SAT* gene family, establishing a foundation for future investigations into the biological functions of *GmSAT* genes and providing valuable insights for soybean genetic improvement strategies.

## Data Availability

All data generated or analyzed in this study are included in the main text and its Supplementary Materials.

## Supplementary Materials

The following supporting information can be downloaded at: https://www.mdpi.com/article/10.3390/ijms26051882/s1.
