# Supplementary material for: Identification and Expression Analysis of the Soybean Serine Acetyltransferase (SAT) Gene Family Under Salt Stress
Source: Int J Mol Sci. 2025 Feb 22;26(5):1882. doi: 10.3390/ijms26051882 (PMC11899845; doi:10.3390/ijms26051882)
Supplement: Supplementary file 1 [file ijms-26-01882-s001.zip › Supplementary Figure.pdf]

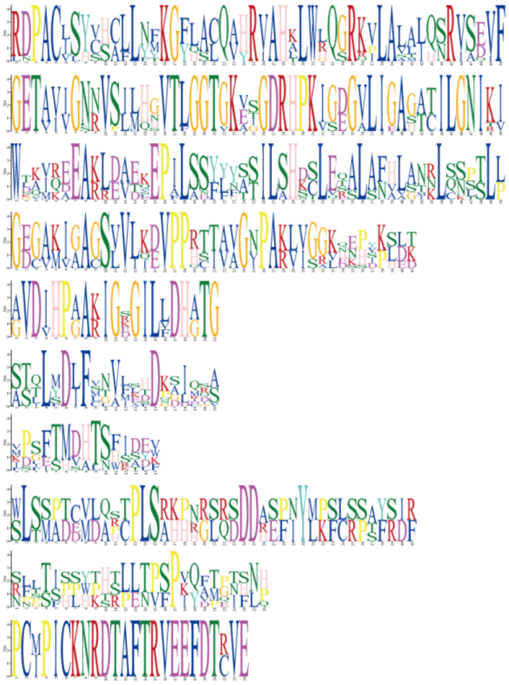

Motif	E-value	Sites	Width
Motif 1	6.2e-284	10	50
Motif 2	6.3e-277	10	50
Motif 3	2.9e-203	10	50
Motif 4	4.0e-157	10	41
Motif 5	1.5e-101	10	21
Motif 6	1.3e-044	10	21
Motif 7	1.5e-028	9	15
Motif 8	1.0e-026	4	41
Motif 9	1.1e-016	6	26
Motif 10	6.3e-010	2	24

Figure S1 Graphical representation of conserved motif amino acids

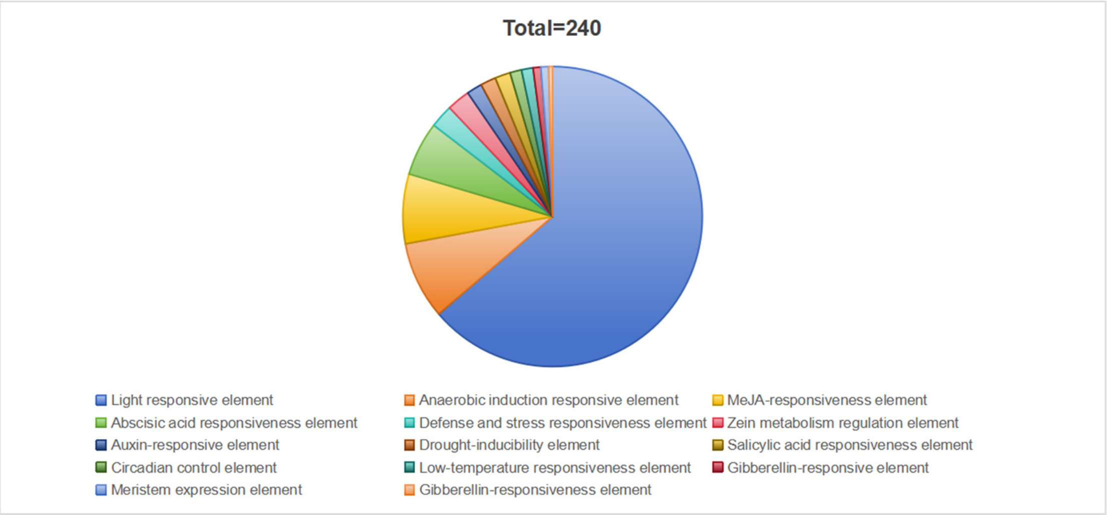

Figure S2 The proportion of various cis-elements
